# Structural similarity assessment for drug sensitivity prediction in cancer

**DOI:** 10.1186/1471-2105-10-S9-S17

**Published:** 2009-09-17

**Authors:** Pavithra Shivakumar, Michael Krauthammer

**Affiliations:** 1 Department of Pathology, Yale University School of Medicine, New Haven, CT, USA

## Abstract

**Background:**

The ability to predict drug sensitivity in cancer is one of the exciting promises of pharmacogenomic research. Several groups have demonstrated the ability to predict drug sensitivity by integrating chemo-sensitivity data and associated gene expression measurements from large anti-cancer drug screens such as NCI-60. The general approach is based on comparing gene expression measurements from sensitive and resistant cancer cell lines and deriving drug sensitivity profiles consisting of lists of genes whose expression is predictive of response to a drug. Importantly, it has been shown that such profiles are generic and can be applied to cancer cell lines that are not part of the anti-cancer screen. However, one limitation is that the profiles can not be generated for untested drugs (i.e., drugs that are not part of an anti-cancer drug screen). In this work, we propose using an existing drug sensitivity profile for drug A as a substitute for an untested drug B given high structural similarities between drugs A and B.

**Results:**

We first show that structural similarity between pairs of compounds in the NCI-60 dataset highly correlates with the similarity between their activities across the cancer cell lines. This result shows that structurally similar drugs can be expected to have a similar effect on cancer cell lines. We next set out to test our hypothesis that we can use existing drug sensitivity profiles as substitute profiles for untested drugs. In a cross-validation experiment, we found that the use of substitute profiles is possible without a significant loss of prediction accuracy if the substitute profile was generated from a compound with high structural similarity to the untested compound.

**Conclusion:**

Anti-cancer drug screens are a valuable resource for generating omics-based drug sensitivity profiles. We show that it is possible to extend the usefulness of existing screens to untested drugs by deriving substitute sensitivity profiles from structurally similar drugs part of the screen.

## Introduction and background

In the last decade, cancer treatment has seen a shift from a "one size fits all" philosophy to a more personalized approach. Technical advances allow for the assessment of complex genetic defects and pathway aberrations, enabling refined cancer classification and treatment prediction based on molecular rather than histological features. Gene expression profiling has been used to classify patient samples as being benign or malignant or to classify them into cancer subclasses [1-3]. Lately, molecular profiling of cancer samples has been applied to the prediction of drug sensitivity [4]. Drug-sensitive samples have been compared to drug-resistant samples to build "drug sensitivity profiles", statistical models built from a defined set of genes whose differential expression in a cell line may confer sensitivity to a drug. Thus, cancer cell lines whose gene expression patterns are similar to the genes in the sensitivity profile will have a higher probability of responding to the drug. It has been demonstrated that gene expression profiling can refine the prediction of drug response to targeted drug therapies. For example, Harris et al. used gene expression profiling to identify lists of genes that are potential predictors of response to Herceptin and Vinorelbine in HER2-positive breast cancers [5]. Another study used the COXEN (CoExpression Extrapolation) algorithm to build sensitivity profiles for the drugs cisplatin and paclitaxel [4]. The profiles were subsequently used to predict drug response in bladder and breast cancer samples. Of particular interest is the fact that these samples were not part of the original anti-cancer cancer panel, showing that drug sensitivity profiles generated from the screen can be generalized to any tumor sample.

Approaches such as COXEN make use of comprehensive pharmacogenomic resources to derive drug sensitivity profiles. COXEN was applied to the NCI-60 (National Cancer Institute) anti-cancer screen, which tested >40,000 compounds on 60 cancer cell lines, all of which have been profiled (in a separate effort) for genome-wide gene expression [6,7]. Sensitivity profiles for the compounds in the screen can be generated by comparing gene expression values of sensitive and resistant cell lines.

The COXEN algorithm (and similar approaches) can only generate profiles for compounds that are part of an anti-cancer drug screen [8]. If a drug was not tested as part of the NCI-60 dataset, its sensitivity profile cannot be generated. Updating an existing cancer screen with the latest available or experimental drugs is a non-trivial issue, and requires the same expertise, infrastructure and conditions as when the screen was established the first time around.

In this work, we present a method to predict responses to drugs that are not part of available anti-cancer screens. We propose to find substitute sensitivity profiles for these untested drugs by using structural similarity.

It has been known that the structure of a compound is related to its activity (QSAR – Quantitative Structure Activity Relationship). Shi et al. performed an analysis where 131 compounds (whose mechanisms of action were known) were clustered based on activity across cancer cell lines [9]. They found that compounds with similar structures clustered together. Several other studies have been performed to show that specific classes of compounds (eg. Taxols) have similar activity patterns across cancer cell lines [10]. Therefore, it can be hypothesized that structurally similar compounds have similar activities and might affect the same pathways. In other words, the sensitivity pattern for drug A (not part of the screen) might be similar to that of drug B (part of the screen) if drug A and drug B are structurally similar. We are interested in extending this idea to test whether the drug sensitivity profiles of a structural analogue of a drug can be used as a substitute when predicting its response in cancer cell lines.

## Results and discussion

To analyze whether a structural analogue of a drug can be used to predict its response, two questions need to be answered. The first question is whether two structurally similar drugs have the same sensitivity pattern across the NCI-60 cancer cell lines. Secondly, even if we can show that there is a strong association between structural similarity and drug sensitivity, we need to test whether the correlation is strong enough to be of any practicality. The second question we would like to ask is whether the sensitivity profile of drug A be used to predict activity of drug B without a significant loss of accuracy, if drug A and drug B are structurally similar. We investigated whether this proposition holds only for compounds in the same chemical family, or also for compounds that are similar but from different families.

### Structural similarity and response similarity in NCI-60 cancer cell lines

The first question we set out to address was whether two structurally similar drugs have the same sensitivity pattern across cancer cell lines in an anti-cancer screen, even when the mechanisms of action or the classes of the compounds might not be known. We used the Tanimoto coefficient between pairs of compounds as the structural similarity measure (see Methods for more details).

**Figure 1:**
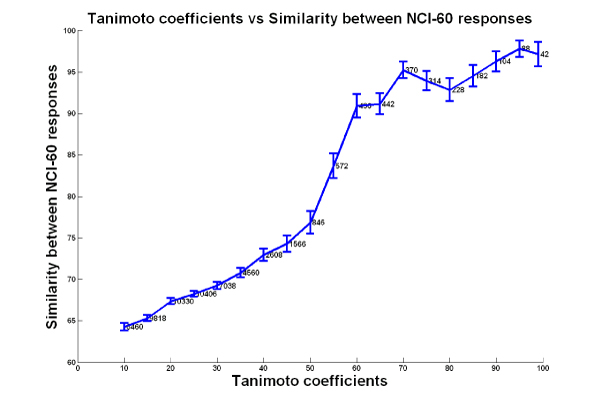
**Tanimoto coefficients vs. Similarity between NCI-60 responses**. Each data point represents a pair of compounds, where the x-coordinate is the Tanimoto coefficient (calculated by Chemcpp), and the y-coordinate is the similarity between their NCI-60 responses. For each interval of Tanimoto coefficients, the 99% confidence intervals (for response similarity values) are shown as the blue bars, with the number of pairs of compounds at each interval indicated.

In figure 1, we show pairs of compounds ordered by their Tanimoto structural similarity coefficients (x-axis), and the similarity between their responses across the 60 cancer cell lines (y-axis). The similarity between responses was measured as the percentage of cell lines that elicited the same response to both the compounds. As the Tanimoto coefficient increases (structures become more similar), the similarity between the cell line responses increases too (i.e., the same set of cell lines are sensitive/resistant to the two compounds). This supports the hypothesis that if two compounds are structurally similar, their sensitivity patterns in the NCI-60 data are similar too. This can be interpreted in the following manner: Two structurally similar drugs may bind similar cellular structures, and activate or inhibit similar cellular pathways, and therefore share a common cause of resistance. We next set out to investigate the practical implications of this finding.

### Predictive power of sensitivity profiles of structurally similar drugs

The second question we wanted to ask is whether we can use one drug as a substitute to perform gene-expression-based sensitivity prediction for another drug. In this scenario, we take advantage of an existing anti-cancer screen, such as the NCI-60, to construct a sensitivity profile for a drug A, and use it as a substitute profile for another drug B. Specifically, we wanted to see what the loss of accuracy in response prediction would be if the sensitivity profile was constructed from a drug with high structural similarity. Using a cross-validation setup within the NCI-60 data set, we could measure the change in prediction accuracy when using drug B's sensitivity profile compared to using a substitute profile generated from drugs A with various levels of structural similarity to B.

**Figure 2:**
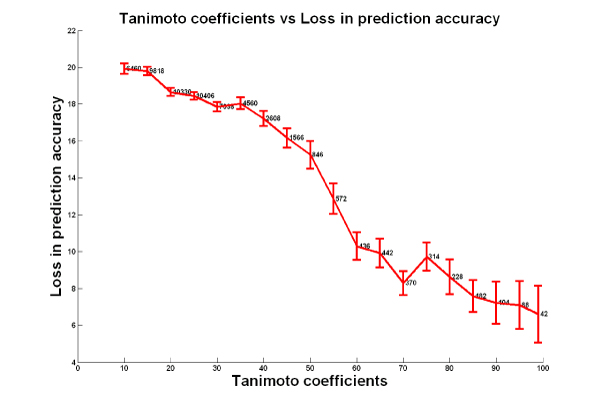
**Tanimoto coefficients vs. Loss in prediction accuracy**. Each data point represents a pair of compounds, where the x-coordinate is the Tanimoto coefficient (calculated by Chemcpp), and the y-coordinate is the loss in prediction accuracy when a substitute drug's sensitivity profile is used in response prediction. For each interval of Tanimoto coefficients, the 99% confidence intervals (for loss in accuracy values) are shown as red bars, with the number of pairs of compounds at each interval indicated.

Figure 2 shows pairs of compounds ordered by their Tanimoto coefficients (x-axis), and the loss of prediction accuracy when one of the pair's sensitivity profile is used as a substitute profile for the other (y-axis). This figure demonstrates that the higher the structural similarity, the lower the loss of accuracy in response prediction. Highly dissimilar compounds (Tanimoto coefficient < 20) show a loss in prediction accuracy of ~20%, while structurally similar compounds (Tanimoto coefficient > 80) show a much lower loss in prediction accuracy (~6%). This drop is highly significant (using a threshold of 80 for the Tanimoto coefficient, the distributions of accuracy values below and above the threshold were significantly different with a p value of <8 × 10^-8^). Therefore, in situations where a drug has not been tested as part of an anti-cancer screen, the sensitivity profile of a structurally similar drug can be used as a substitute in response prediction without a significant loss in accuracy.

### Families of compounds in our dataset

**Figure 3:**
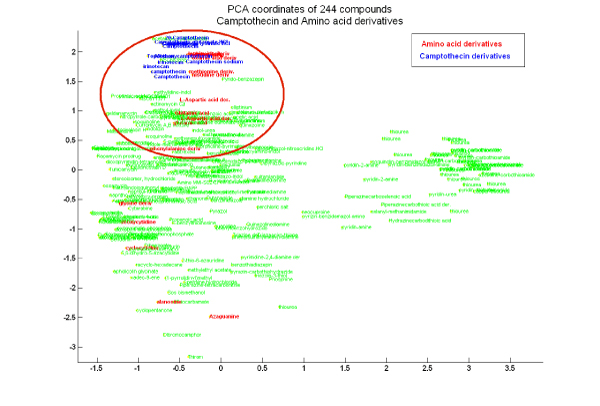
**Visualization of the clustering of compounds in the NCI-60 data**. The first two principal components of each of the 244 compounds are used as x and y coordinates to visualize the clustering of the compounds. Compounds closer to each other in the plot represent structurally similar compounds. Amino acid derivatives are highlighted in red, while derivatives of camptothecin are highlighted in blue, while all other compounds are shown in green.

To ensure that not all structurally similar pairs of compounds in our dataset belonged to the same family, we identified 1130 pairs of compounds with Tanimoto coefficients higher than 70. We were able to extract several pairs of interest where the compounds did not belong to the same family. Figure 3 shows derivatives of Camptothecin (a Topoisomerase I inhibitor [11]) along with amino acid derivatives clustered together in a visualization of the 244 compounds in our dataset. Comparing their activity patterns in NCI-60, we found that both classes exhibited exactly the same activity across the cell lines. Both camptothecins and amino-acid derivatives have been known to have anti-neoplastic activities, but we couldn't find any evidence in the literature of the amino acid derivatives inhibiting Topoisomerase I. The above example shows that while the pairs of compounds might belong to different functional or mechanistic families, structural similarities (> 70%) highly correlate with their cellular responses.

## Conclusion

Cancer patients need access to the latest decision criteria given an increasing number of highly specific and targeted anti-cancer drugs. It is believed that the pre-treatment cellular state is predictive of whether a cancer cell is going to respond to a particular drug. Functional anti-cancer drug screens give access to pre-treatment gene expression states from sensitive and resistant cancer cells, and are useful in deriving drug sensitivity profiles that can be applied to independent cancer samples. One major limitation of this approach is that profiles can only be generated for drugs that have been tested in anti-cancer screens. In our work, we addressed the issue of deriving sensitivity profiles for untested drugs for which we do not (yet) have response information across a number of cell lines. For those drugs, we examined the possibility of using substitute drug sensitivity profiles from structurally similar drugs that have been tested in the screen.

We established that it is indeed true that structural similarity is highly correlated with sensitivity across cancer cell lines. We have also shown that for structurally related drugs, the use of substitute sensitivity profiles is likely to result in response predictions similar to using a drug's own profile in the first place. Our study may thus be helpful in increasing the relevance of existing screens, by expanding their use to other drugs (not part of the screen) with high structural similarity. Since not all new cancer drugs are immediately tested in anti-cancer screens, our approach ultimately increases the number of drugs for which patient response can be predicted.

A drawback of the current study is the small number of drugs that we could include in our analysis, i.e. drugs that showed sufficient variation across cell lines. This introduces some bias that may favor pairs of compounds in the same class and with high similarity in activity patterns. Nonetheless, the above experiments demonstrate that there is a strong correlation between structural similarity and drug sensitivity in the NCI-60 cancer cell lines.

In terms of future work, we want to explore the following issues. First, we want to fine-tune our sensitivity profile generating algorithm, in order to increase the prediction accuracy when using a compound's own profile (which currently averages at 74%). Second, we want to explore other structural similarity measures to ensure that we indeed identify the closest structural pairs among the NCI-60 compounds. Third, we want to investigate other similarity measures (based on function or mechanism) that can identify an analogue for sensitivity profile substitution.

## Methods

### NCI-60 datasets

The NCI-60 is an anti-cancer screen composed of over 40,000 compounds tested on 60 cancer cell lines from 9 different tissue types. We downloaded the compounds' log(GI_50_) values (concentration of compound needed for 50% growth inhibition) from . Gene expression values for the 60 untreated cancer cell lines were obtained from Gene Expression Omnibus (Accession GDS1761) [7].

Compound selection criteria were adopted from Staunton et al [8]. Briefly, for each compound, the 60 log(GI_50_) values (representing the responses of the 60 cancer cell lines to the compound) were normalized. Those cell lines with log(GI_50_) values that were at least 0.8 standard deviations above the mean were considered sensitive to the compound, and those values at least 0.8 standard deviations below the mean were considered resistant. Log(GI_50_) values that fell within the 1.6 standard deviation window around the mean were considered intermediate, and were not included in the analysis. Since only compounds that exhibit a variable response across the cancer cell lines are useful in sensitivity analysis, the 40,000 compounds were filtered in the following manner -

1. Standard deviation of the log(GI_50_) values of the compound across the 60 cancer cell lines is at least 0.625

2. At least 10 of the 60 cell lines are sensitive

3. At least 10 of the 60 cell lines are resistant

4. At least 30 of the 60 cell lines are either sensitive or resistant (do not fall in the intermediate category)

244 compounds matched the aforementioned criteria, and were used in our analysis.

### Structural similarity and response similarity in NCI-60 cancer cell lines

#### Structural similarity between compounds

Swamidass et al. evaluated different structural similarity metrics (including the Tanimoto coefficient) to classify compounds as being sensitive or resistant to the NCI-60 cell lines [12]. They concluded that 2D representations of molecules yield the best accuracies when assigning the response class of a cell line to a compound. Therefore, we used the Tanimoto coefficients on the 2D representations of the compounds when calculating their pair-wise similarities. To compute the Tanimoto coefficient between all pairs of the 244 compounds in our dataset, we used Chemcpp (a chemoinformatics toolbox that computes similarity between chemical compounds), available at[13]. Each compound's 2D structure, represented as a mol file, was downloaded from the Pubchem server [14]. Briefly, the Tanimoto coefficient represents the similarity between two compounds based on the presence or absence of molecular fragments. Chemcpp uses a graph-based approach to compute the Tanimoto kernel between two molecules as follows



where P is the set of molecular fragments taken into account, and *ϕ*_*G*_(*p*) is a bit value representing the presence of a molecular fragment p in the graph representation of a compound, G. Chemcpp outputs the normalized Tanimoto coefficients between all pairs of compounds in the dataset, thus giving a relative measure of similarity among them.

#### Response similarity between compounds

For each pair of compounds in our dataset, the similarity between their responses across the 60 cell lines was calculated as the percentage of cell lines that responded similarly to (either both sensitive or both resistant) both the compounds. Cell lines classified as intermediate for either of the compounds were not included in the calculation. Figure 1 shows Tanimoto coefficients plotted along response similarities, to demonstrate the correlation between structural similarity and response similarity in the NCI-60 cancer cell lines.

### Predictive power of sensitivity profiles of structurally similar drugs

If a drug has not been tested as part of a screen, we wanted to see how much accuracy would have to be compromised if a structurally similar drug was used as a substitute to predict response. This second question involved generating the sensitivity profiles for each of the 244 compounds in our dataset. We then used the sensitivity profile of a drug A as a substitute for drug B, recorded the structural similarity between A and B, and measured the drop in prediction accuracy compared to using drug B's sensitivity profile.

#### Sensitivity profile generating algorithm

For each of the 244 compounds, the log(GI_50_) data and gene expression data were integrated to generate sensitivity profiles (a statistical model based on a list of differentially expressed genes) which were then used to predict the response of a cancer cell line to one of the compounds. For each compound, the response of each of the cell lines in the NCI-60 panel was predicted in a leave-one-out cross-validation setting. Specifically, the remaining 59 cell lines were used as a training set to predict the response of the compound to the 60th cell line using a generalized linear model. The steps of our algorithm were as follows

For each of the compounds:

1. Using each one of the 60 cell lines as the testing set, the remaining 59 cell lines were assigned as the training set.

2. The 59 cell lines were separated into those sensitive and resistant to the compound.

3. Using the expression values of 9706 genes across the cell lines, a t-test was used to identify the 10 most differentially expressed genes between the sensitive and resistant sets. These 10 genes were identified as the 'sensitivity profile' genes for the compound.

4. A generalized linear model was used in Matlab to predict the response of the 60th cell line, using the expression values of the 10 genes that were part of the sensitivity profile [15].

#### Structural similarity and sensitivity prediction

For each pair of drugs A and B for which the Tanimoto coefficient was available, the loss of prediction accuracy was calculated in the following manner

1. The sensitivity profile was generated for all 244 compounds using NCI-60 data.

2. For each drug A, 60 responses were predicted for the NCI-60 cell lines using the drug's profile. The predicted responses were compared to drug A's experimental responses from NCI-60 to calculate an overall accuracy for drug A, which we termed Accuracy_A using A's profile_

3. The profiles of the remaining 243 drugs (drug B) were used to predict responses of drug A to the 60 cancer cell lines. These predicted responses were compared to drug A's experimental responses to calculate Accuracy_A using B's profile_

4. The loss in prediction accuracy was calculated as the absolute difference between Accuracy_A using A's profile _and Accuracy_A using B's profile_. This difference can be thought to represent the loss of accuracy when using another drug's profile as a substitute in response prediction

Figure 2 shows Tanimoto coefficients plotted along loss in prediction accuracies, to demonstrate the relationship between structural similarity and predictive value of substitutive sensitivity profiles in the NCI-60 dataset.

### Families of compounds in our dataset

To analyze the families of the compounds in our dataset, we first annotated all 244 compounds with their generic or IUPAC names, downloaded from Pubchem [5]. Using these annotations, we were able to identify broad families for most of the compounds. Using the Tanimoto structural similarity matrix calculated by Chemcpp, we calculated the first two principal components of each of the compounds, using Matlab's princomp function. We used these two components as the x and y coordinates for visualizing the clustering of the compounds in Figure 3.

## Competing interests

The authors declare that they have no competing interests.

## Authors' contributions

PS carried out the computational work, and took the lead in writing the paper. MK supervised PS, and was involved in drafting the manuscript. All authors read and approved the final manuscript.
